# Association between the dietary inflammatory index and depressive symptoms in adults with cardiovascular–kidney–metabolic syndrome: evidence of metabolic syndrome as a mediator

**DOI:** 10.3389/fnut.2025.1623482

**Published:** 2025-07-30

**Authors:** Ruo-Nan Tian, Sheng-Xiao Zhang, Nan Zhang, Wen-Jing Wu, Hua-Qing Guo, Chen Wang, Zhi-Guang Duan

**Affiliations:** ^1^College of Humanities and Social Sciences, Shanxi Medical University, Taiyuan, Shanxi, China; ^2^Key Laboratory of Cellular Physiology at Shanxi Medical University, Ministry of Education, Taiyuan, Shanxi, China; ^3^Shanxi Provincial Key Laboratory of Rheumatism Immune Microecology, Taiyuan, Shanxi, China; ^4^Department of Rheumatology and Immunology, The Second Hospital of Shanxi Medical University, Taiyuan, Shanxi, China; ^5^SXMU-Tsinghua Collaborative Innovation Center for Frontier Medicine, Shanxi Medical University, Taiyuan, Shanxi, China; ^6^School of Management, Shanxi Medical University, Taiyuan, Shanxi, China

**Keywords:** depressive symptoms, cardiovascular-kidney-metabolic syndrome, diet, DII, adult population

## Abstract

**Background:**

This study aims to investigate the association between the dietary inflammatory index (DII) and depressive symptoms (Deps) in adults with cardiovascular–kidney–metabolic (CKM).

**Methods:**

Using data from the National Health and Nutrition Examination Survey (NHANES) spanning from 2005 to 2018, we examined the association between DII and Deps in CKM patients. We employed weighted multivariate logistic regression, generalized additive models, and restricted cubic spline models. Threshold effect analysis was performed to identify the inflection point in the smooth curves. Subgroup and sensitivity analyses were conducted to validate the robustness of the findings. Additionally, we used the Bootstrap method to evaluate the mediating role of metabolic syndrome (Mets).

**Results:**

A total of 12,980 participants were included, with 1,096 (8.4%) identified as experiencing Deps. DII was associated with the risk of Deps, which remained robust after adjusting for 20 potential confounders. Specifically, each unit increase in DII was associated with an 18.7% higher incidence of Deps. The relationship between DII and Deps in CKM patients exhibited a J-shaped pattern, with a non-linear positive correlation observed in the Non-advanced CKM population. Subgroup analyses confirmed the correlation. Additionally, Mets mediated 4.44% of the observed effect.

**Conclusion:**

The DII was non-linearly associated with the risk of Deps in CKM patients. This highlights the importance of targeted interventions to address this comorbidity and mitigate the disease burden associated with both Deps and CKM syndrome.

## 1 Introduction

Cardiovascular-kidney-metabolic (CKM) syndrome, recently defined by the American Heart Association (AHA), describes the interplay among obesity, diabetes, chronic kidney disease (CKD), and cardiovascular disease (CVD) (1). Based on risk factors and diagnosed disease, the stages range from 0 (no risk factors) to 4 (established CVD) (1). Recent studies have highlighted the high global prevalence of CKM syndrome (2), posing a significant burden on global public health systems and socioeconomics (3). Low mood represents the central clinical aspect of depression. The World Health Organization (WHO) has projected that by 2030, depression will become the third leading contributor to global disease burden (4). Both CKM syndrome and depressive symptoms (Deps) pose major public health challenges. Metabolic disease (5), CVD (6), and CKD (7) are independently associated with depression. A recent prospective cohort study showed that CKM was independently linked to an increased risk of depression, with this relationship becoming more pronounced as CKM staging progressed through advanced stages (8).

Dietary inflammatory index (DII) serves as a critical tool for assessing the overall inflammatory potential of diet by evaluating the inflammatory properties of individual dietary components (9). A higher DII score indicates a greater pro-inflammatory potential, which correlates with six key inflammatory biomarkers: Interleukin-1β (IL-1β), Interleukin-4 (IL-4), Interleukin-6 (IL-6), Interleukin-10 (IL-10), Tumor Necrosis Factor-α (TNF-α), and C-reactive protein (CRP) (9, 10). The DII has been widely utilized to explore the relationship between dietary patterns and various chronic diseases, with unhealthy diets recognized as significant risk factors for these conditions. Numerous studies have highlighted the pivotal role of dietary intake in the development of chronic inflammation. For instance, diets high in saturated fats and red meat are associated with elevated inflammatory markers, which increase the risk of obesity, type 2 diabetes (T2DM), CVD, and CKD (11, 12), ultimately contributing to the onset and progression of CKM. In contrast, the Mediterranean diet, rich in whole grains, vegetables, and healthy fats, is related to reduced inflammation levels (13), offering a protective effect against CKM development. Several studies have demonstrated that DII scores are associated with key components of CKM, including metabolic diseases (14), diabetes (14), obesity (14), CVD (15), and CKD (16).

While the etiology of depression remains incompletely understood, available research suggests that depression episodes are often accompanied by altered inflammatory states (17, 18). A meta-analysis showed that nearly one in four individuals with Deps exhibited signs of low-grade inflammation, with 58% showing mildly elevated CRP levels (19). Moreover, dietary components, including specific foods and nutrients, possess pro-and anti-inflammatory properties that may influence depression (20). However, it remains unclear whether certain dietary patterns may exacerbate Deps in people with CKM. To address this gap, we utilized data from the National Health and Nutrition Examination Survey (NHANES) spanning from 2005 to 2018 to investigate the relationship between the DII and the risk of Deps in U. S. adults with CKM.

## 2 Methods

### 2.1 Data source and study population

The NHANES is a study designed to evaluate the health and nutritional status of the civilian non-institutionalized population in the United States. It employs a multistage sampling strategy to collect interview, examination, and laboratory data from participants. The NHANES protocol is approved by the NCHS Research Ethics Review Board, and informed consent is obtained from each participant. Data are publicly accessible via the NHANES website^1^ (21).

**Figure 1:**
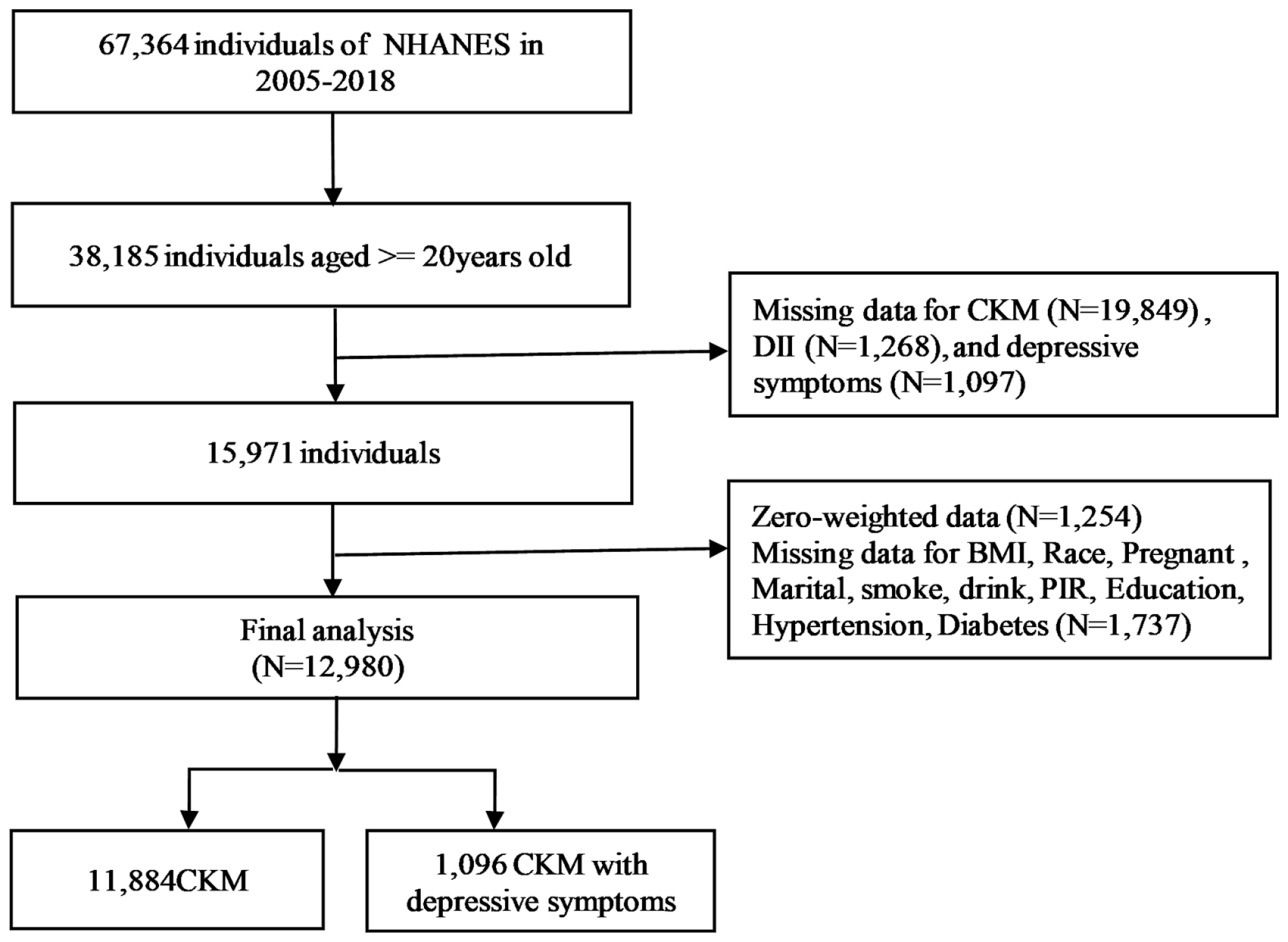
Flowchart of the study population.

This study utilized NHANES data from the pre-pandemic period, encompassing 67,364 participants. The inclusion criteria were adults aged 20 years or older. Participants were excluded if they had incomplete dietary information, missing data from the Deps questionnaire, or insufficient information to assess the presence of CKM syndrome. The selection process is depicted in Figure 1.

### 2.2 The definition of DII

NHANES collects dietary data through 24-h dietary recall interviews for the calculation of DII scores. The DII, a scoring system developed by Shivappa et al. (9), is a widely used metric for assessing overall dietary inflammatory potential and encompasses 45 dietary parameters. DII scores remain valid even when fewer than 30 food parameters are included (22, 23). Due to the limitations in the range of dietary components assessed in NHANES, and in reference to prior studies, 28 food parameters were incorporated into the DII calculation. All food ingredient-specific DII scores are summed to produce an overall DII, where positive scores (>0) reflect pro-inflammatory potential, negative scores (<0) indicate anti-inflammatory potential, and zero scores represent no significant effect on inflammatory potential.

DII = (*Z* score × the inflammatory effect score of each dietary component).

*Z* score = (daily mean intake – global daily mean intake)/standard deviation.

*Z* score = Z score → (converted to a percentile score) × 2–1.

### 2.3 The definition of CKM syndrome

Cardiovascular–kidney–metabolic syndrome is a systemic health disorder caused by pathophysiological interactions between obesity, T2DM, CKD, and CVD (1). It is categorized into five stages. Specifically, Stage 0 is characterized by the absence of CKM risk factors. Stage 1 includes individuals with elevated body mass index (BMI), increased waist circumference, or prediabetes. Stage 2 encompasses participants with metabolic risk factors or moderate-to-high-risk CKD, as per Kidney Disease Improving Global Outcomes (KDIGO) criteria (1). Stage 3 is identified based on the presence of very-high-risk KDIGO CKD stages (1) or a high-predicted 10-year CVD risk. 10-year cardiovascular risk is estimated using the AHA Predicting Risk of CVD EVENTs (PREVENT) equations (24). Stage 4 involves individuals with self-reported established cardiovascular disease (coronary heart disease, angina, heart attack, heart failure, and stroke). Stages 3 and 4 are collectively classified as advanced CKM syndrome, encompassing individuals diagnosed with or at high risk of developing cardiovascular disease (22). Detailed descriptions of stage definitions are shown in Supplementary Table S1.

### 2.4 The definition of deps

NHANES utilizes the validated Patient Health Questionnaire (PHQ-9) to assess Deps. The PHQ-9 is designed to evaluate participants’ depressive symptoms over the past 2 weeks, including indicators such as insomnia, reduced appetite, and feelings of loneliness. Responses to the nine symptom-related questions are categorized as “Not at all,” “Several days,” “More than half the days,” and “Nearly every day,” with corresponding scores ranging from 0 to 3. The total score ranges from 0 to 27 points. A PHQ-9 score of 10 or higher is defined as indicative of Deps (23). This study will analyze the odds of Deps and use the total PHQ-9 score as the outcome variable.

### 2.5 Covariates

Based on previously published literature, potential covariates that may affect dietary quality and Deps were selected, including demographic and health-related factors. These covariates were composed of gender, age, race, education, family income-to-poverty ratio (PIR), marital status, BMI, smoking status, hypertension, diabetes, and physical activity (25, 26) (PA, MET-min/week). Alcohol consumption was included in the DII and, therefore, was not considered separately. For detailed definitions and categorizations of the covariates, please refer to Supplementary Table S2.

### 2.6 Statistical analysis

We accounted for the complex sampling design and applied appropriate weights following the NHANES Analytic Guidelines. The sampling weight was calculated using the formula: WS14YR = 1/7* SAF2YR (SAF2YR is the 2-year sample weight in each survey period; WS14YR is the sample weight calculated after combining the seven periods). Continuous variables were analyzed using the Student’s *t*-test for complex survey samples, while categorical variables were analyzed using the chi-square test. Participant characteristics were analyzed by DII quartiles, with continuous variables presented as means and standard errors (SE), and categorical variables as counts and percentages (%).

DII was explored both as a continuous variable (with per 10-point increment) and a categorical variable (tertiles). We employed weighted multivariate logistic regression models to explore the relationship between CKM and DII. We adjusted for various potential confounders. Model 1 was unadjusted. Model 2 adjusted for age, race, and gender. Model 3 was fully adjusted for all covariates in addition to those included in Model 2. The generalized additive model (GAM) and restricted cubic spline (RCS) analysis were employed to assess potential non-linear associations between DII and Deps at different CKM stages. The piecewise regression model and logarithmic likelihood ratio test were used to analyze the threshold effect, aiming to find the turning point of the curve and then analyze the effect piecewise.

To determine whether metabolic syndrome (Mets) acts as a mediator in the relationship between DII and Deps in participants with CKM, we utilized the R package “mediation” and performed 1,000 bootstrap simulations to estimate the mediation effects of each mediator and calculate the proportion of mediation. The direct effect (DE) refers to the influence of DII on depressive symptoms without mediation, while the indirect effect (IE) represents the effect of DII on Deps via mediation. The ratio of the IE to the total effect (TE) was used to represent the magnitude of the mediation effect.

We performed subgroup analyses, stratified by confounders, and assessed their interactions to ensure consistency of results across demographic and clinical subgroups. Sensitivity analyses were conducted by excluding obese (BMI ≥ 35.0) participants. In addition, unweighted logistic analyses and multiple interpolation using chained equations were employed to address missing data for covariates. All statistical analyses were performed with R (version 4.3.1) and a two-sided *p*-value less than 0.05 was deemed statistically significant.

## 3 Results

### 3.1 Baseline characteristics of participants

A total of 12,980 participants were included from the NHANES, representing approximately 188.7 million non-institutionalized US residents. The baseline characteristics of the study population, stratified by Deps, are presented in Table 1. Among the participants, 1,096 had Deps and CKM (8.443%). Deps was more prevalent in females (63.925%) than in males (36.075%). Compared to participants with CKM who did not exhibit Deps, those with Deps were mostly middle-aged (40–59 years), non-Hispanic Black, highly educated (beyond college level), unmarried or without a partner, obese, had a lower family PIR, and engaged in low-intensity physical activity (PA). Furthermore, fewer participants had diabetes and hypertension, and smoked. However, no significant differences were observed between the groups in terms of age (*p* = 0.491) and PA (*p* = 0.141).

**Table 1:** General characteristics of participants in NHANES 2005–2018.

Characteristics	Total	CKM without Deps	CKM with Deps	*p*-value
	*N* = 12,980	*N* = 11,884	*N* = 1,096	
Gender, *n* (%)				<0.001
Female	6,494 (50.566)	5,789 (49.518)	705 (63.925)	
Male	6,486 (49.434)	6,095 (50.482)	391 (36.075)	
Age, (years), mean ± SD	47.390 ± 0.273	47.357 ± 0.293	47.805 ± 0.571	0.491
Age group, *n* (%)				<0.001
20–39	4,180 (36.182)	3,875 (36.556)	305 (31.422)	
40–59	4,380 (37.762)	3,928 (37.189)	452 (45.065)	
>=60	4,420 (26.056)	4,081 (26.255)	339 (23.513)	
Race, *n* (%)				0.001
Mexican American	1979 (7.859)	1822 (7.935)	157 (6.887)	
Non-Hispanic White	2,563 (10.545)	2,326 (10.273)	237 (14.006)	
Non-Hispanic Black	5,928 (69.746)	5,429 (70.076)	499 (65.540)	
Others	2,510 (11.850)	2,307 (11.716)	203 (13.567)	
Marital, *n* (%)				<0.001
Married or partnered	7,876 (64.190)	7,386 (65.470)	490 (47.873)	
Not married or no partner	5,104 (35.810)	4,498 (34.530)	606 (52.127)	
Education, *n* (%)				<0.001
Under high school	2,959 (14.978)	2,580 (14.188)	379 (25.047)	
High school or equivalent	3,009 (23.444)	2,734 (23.104)	275 (27.778)	
College or higher	7,012 (61.578)	6,570 (62.709)	442 (47.175)	
BMI (kg/m^2^), *n* (%)				<0.001
Underweight	195 (1.579)	173 (1.550)	22 (1.943)	
Normal	3,498 (28.362)	3,258 (28.600)	240 (25.335)	
Obese	4,997 (37.385)	4,444 (36.504)	553 (48.610)	
Overweight	4,290 (32.674)	4,009 (33.346)	281 (24.112)	
PIR, *n* (%)				<0.001
0–1.29	3,910 (20.423)	3,316 (18.677)	594 (42.670)	
1.30–3.49	5,013 (36.781)	4,663 (36.930)	350 (34.889)	
≥3.50	4,057 (42.796)	3,905 (44.393)	152 (22.441)	
PA, *n* (%)				0.141
No physical activity	204 (1.602)	186 (1.948)	18 (2.976)	
Low intensity physical activity	5,747 (46.698)	5,330 (58.844)	417 (55.763)	
High intensity physical activity	3,825 (31.318)	3,544 (39.208)	281 (41.261)	
Diabetes, *n* (%)				<0.001
No	11,120 (89.674)	10,260 (90.130)	860 (83.870)	
Yes	1860 (10.326)	1,624 (9.870)	236 (16.130)	
Hypertension, *n* (%)				<0.001
No	7,386 (61.894)	6,870 (62.699)	516 (51.649)	
Yes	5,594 (38.106)	5,014 (37.301)	580 (48.351)	
Smoke, *n* (%)				<0.001
No	10,366 (80.011)	9,669 (81.543)	697 (60.497)	
Yes	2,614 (19.989)	2,215 (18.457)	399 (39.503)	
CKM stages, *n* (%)				<0.001
Non-advanced stages (stages 0–2)	10,719 (87.136)	9,909 (87.724)	810 (79.657)	
Advanced stages (stages 3 or 4)	2,261 (12.864)	1975 (12.276)	286 (20.343)	

### 3.2 Association between DII and deps in participants with CKM

**Figure 2:**
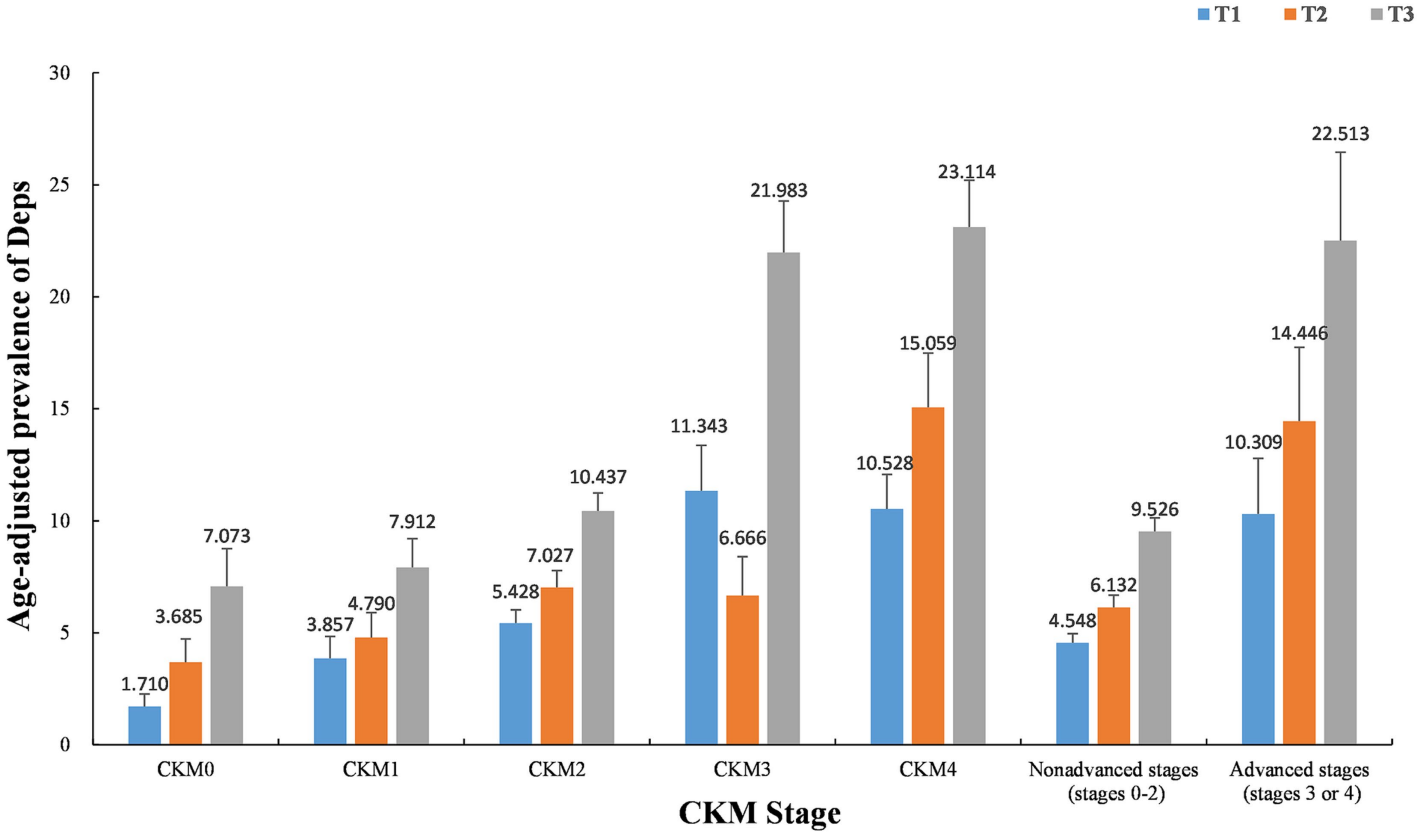
Age-adjusted prevalence of Deps in different levels of DII scores. Numbers at the top of the bars represent the weighted percentage. Bar whiskers represent the SE. DII dietary inflammatory index. DII was converted from a continuous variable to a categorical variable: T1, 1st tertile; T2, 2nd tertile; T3, 3rd tertile.

Figure 2 illustrates the age-adjusted prevalence of Deps among participants across different stages of CKM. Participants with high DII levels in CKM stage 0–4 exhibited a higher age-adjusted prevalence of Deps compared to those with moderate and low DII levels. Furthermore, an increasing trend in Deps prevalence was observed as the severity of CKM escalated among participants at the same DII level. Table 2 illustrates the comparison of DII component scores between the CKM group with Deps and the CKM group without Deps.

**Table 2:** Comparison of DII component scores between the CKM with Deps group and the CKM group.

Characteristics	Total	CKM	CKM with Deps	*P*-value
	*N* = 12,980	*N* = 11,884	*N* = 1,096	
DII	1.445 (0.033)	1.398 (0.034)	2.046 (0.069)	<0.001
Energy	−0.001 (0.002)	0.001 (0.002)	−0.026 (0.006)	<0.001
Protein	−0.001 (0.000)	0.000 (0.000)	−0.005 (0.001)	<0.001
Carbohydrate	−0.021 (0.001)	−0.021 (0.001)	−0.027 (0.003)	0.046
Fiber	0.186 (0.008)	0.178 (0.009)	0.296 (0.016)	<0.001
Total fat	0.039 (0.003)	0.043 (0.003)	−0.001 (0.009)	<0.001
Saturated fat	−0.056 (0.003)	−0.053 (0.003)	−0.090 (0.010)	<0.001
MUFA	0.000 (0.000)	0.000 (0.000)	0.001 (0.000)	<0.001
PUFA	−0.082 (0.003)	−0.085 (0.003)	−0.031 (0.011)	<0.001
Cholesterol	−0.018 (0.001)	−0.017 (0.001)	−0.031 (0.004)	<0.001
β-Carotene	0.342 (0.005)	0.338 (0.005)	0.400 (0.013)	<0.001
Niacin	0.017 (0.002)	0.014 (0.002)	0.052 (0.006)	<0.001
Vitamin A	0.187 (0.003)	0.184 (0.003)	0.224 (0.007)	<0.001
Vitamin B1	0.013 (0.001)	0.012 (0.001)	0.024 (0.002)	<0.001
Vitamin B2	−0.014 (0.001)	−0.015 (0.001)	−0.005 (0.002)	<0.001
Vitamin B6	−0.090 (0.003)	−0.095 (0.003)	−0.027 (0.009)	<0.001
Vitamin B12	−0.017 (0.001)	−0.016 (0.001)	−0.028 (0.003)	<0.001
Vitamin C	0.214 (0.004)	0.212 (0.004)	0.246 (0.012)	0.007
Vitamin D	0.214 (0.004)	0.212 (0.004)	0.242 (0.011)	0.011
Vitamin E	0.101 (0.005)	0.097 (0.005)	0.153 (0.013)	<0.001
Folic acid	0.105 (0.002)	0.104 (0.002)	0.116 (0.004)	0.017
Mg	0.041 (0.005)	0.035 (0.005)	0.116 (0.011)	<0.001
Fe	0.001 (0.000)	0.001 (0.000)	−0.005 (0.001)	<0.001
Zinc	−0.022 (0.004)	−0.027 (0.003)	0.046 (0.010)	<0.001
Selenium	−0.103 (0.001)	−0.106 (0.001)	−0.064 (0.005)	<0.001
Caffeine	0.084 (0.000)	0.084 (0.000)	0.084 (0.000)	0.091
Alcohol	0.154 (0.003)	0.152 (0.004)	0.182 (0.009)	0.004
*n*-3 Fatty acids	0.266 (0.001)	0.265 (0.001)	0.275 (0.003)	<0.001
*n*-6 Fatty acids	−0.065 (0.001)	−0.067 (0.001)	−0.046 (0.004)	<0.001

Table 3 presents the association between DII and Deps in participants with CKM. Weighted multivariate logistic regression analysis was conducted to assess the relationship between DII and the presence of Deps. In continuous models, both the unadjusted model (OR = 1.223; 95% CI: 1.164–1.285; *p* < 0.001) and the minimally adjusted model (OR = 1.187; 95% CI: 1.129, 1.247; *p* < 0.001) demonstrated a positive association between DII and Deps in participants with CKM. Specifically, each unit increase in DII was associated with an 18.7% higher risk of experiencing Deps. This relationship persisted and remained robust in the fully adjusted model (OR = 1.078; 95% CI: 1.018–1.142; *p* = 0.011). In the categorical model, where DII was categorized into tertiles, higher DII scores were associated with an increased risk of Deps in CKM participants. Specifically, compared to the T1 group, the odds ratio for the T3 group was 2.226 (95% CI: 1.818–2.725, *p* < 0.001). After fully adjusting for potential confounders, the odds of Deps remained significantly elevated in T3 group compared to the T1 group (T3: OR = 1.321, 95% CI: 1.018–1.714, *p* = 0.036).

**Table 3:** Logistic regression on the association between DII and Deps in participants with CKM.

Characteristics	Model 1	*p*-value	*p* for trend	Model 2	*p*-value	*p* for trend	Model 3	*p*-value	*p* for trend
	OR [95%CL]			OR [95%CL]			OR [95%CL]		
DII	1.223 (1.164,1.285)	<0.001	–	1.187 (1.129,1.247)	<0.001	–	1.078 (1.018,1.142)	0.011	–
DII [Per 10-point increase]	7.480 (4.549,12.301)	<0.001		5.535 (3.357,9.128)	<0.001		2.252 (1.397,3.631)	0.001	
Categories DII									
T1	1[Ref]	Ref	<0.001	1[Ref]	1[Ref]	<0.001	1[Ref]	1[Ref]	0.310
T2	1.341 (1.056,1.704)	0.017		1.250 (0.982,1.591)	0.070		1.158 (0.848,1.580)	0.353	
T3	2.226 (1.818,2.725)	<0.001		1.954 (1.586,2.409)	<0.001		1.321 (1.018,1.714)	0.036	

Data are presented as OR (95% CI).

Model 1, unadjusted covariates. Model 2, adjusted for gender, age and race. Model 3, PIR, BMI, marital, education, diabetes, hypertension and smoke were further adjusted based on the previous model.

DII was converted from a continuous variable to a categorical variable: T1, 1st tertile; T2, 2nd tertile; T3, 3rd tertile.

### 3.3 Smooth curve fitting and threshold effect analysis

**Figure 3:**
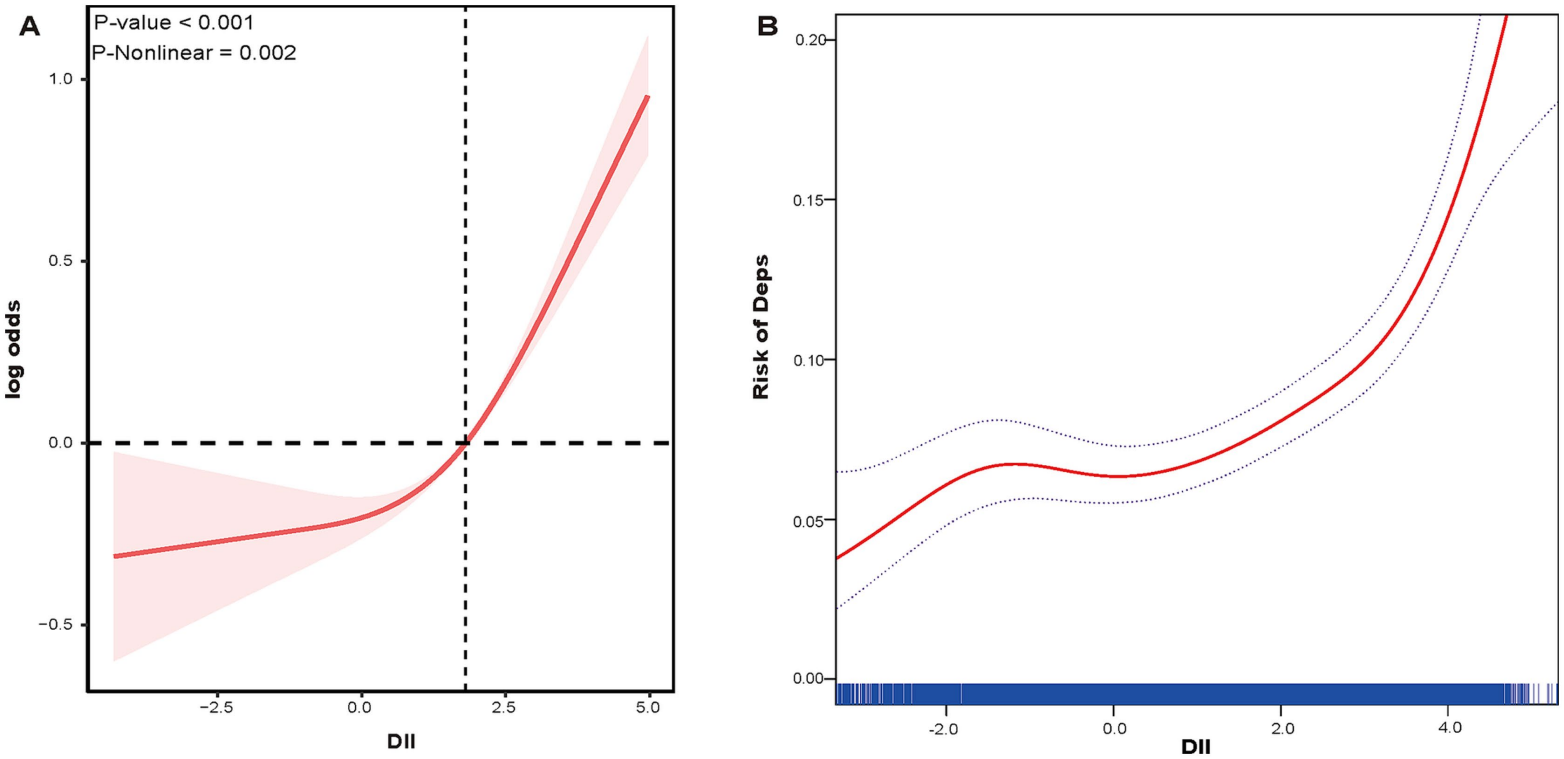
The RCS curve of the association between DII and Deps among CKM participants **(A)**. Smooth curve plot: DII levels and Deps among CKM participants **(B)**. The red curve is the actual fitted curve, and the upper and lower two blue dashed lines represent the 95% confidence interval.

A three-section restricted cubic spline analysis revealed a non-linear dose–response relationship between DII and the risk of Deps (*P* for non-linearity = 0.002, Figure 3A). Figure 3B illustrates the results of the smoothed curve fitting based on the GAMs. Both analyses demonstrated a J-shaped relationship, reaching a turning point at which an increase in DII led to a rapid increase in the risk of Deps. The maximum likelihood method was used to identify the turning point of the smoothed curve, which occurred at DII = 3.285. The results are shown in Table 4.

**Table 4:** Threshold effect of DII levels on Deps in participants with CKM.

DII	β-coefficients (95%CI)	*P*-value	Log likelihood ratio test
All data	0.161 (0.045, 0.080)	<0.001	0.002
<3.285	0.114 (1.057, 1.187)	<0.001	
>=3.285	0.576 (1.389, 2.279)	<0.001	

**Figure 4:**
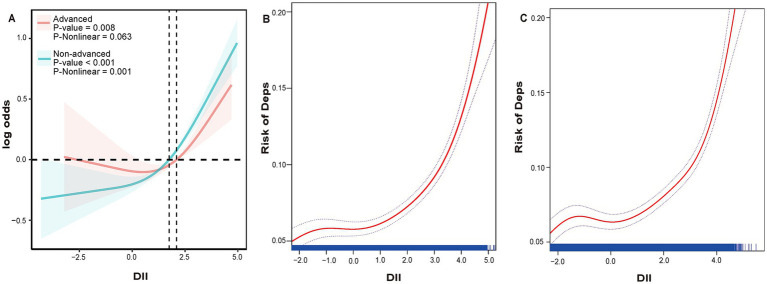
The RCS curve of the association between DII and Deps among CKM participants which were categorized by stage **(A)**. Smooth curve plot: DII levels and Deps among Non-advanced CKM participants **(B)** and advanced CKM participants **(C)**. The red curve is the actual fitted curve, and the upper and lower two blue dashed lines represent the 95% confidence interval.

Furthermore, Figure 4A reveals the non-linear dose–response relationship between DII and risk of Deps for participants at different stages of CKM using a three-section restricted cubic spline analysis. Figures 4B,C show the results of smoothed curve fitting based on the GAMs for participants at different CKM stages. Notably, individuals in the advanced stage of CKM showed no significant non-linear relationship (*P* for non-linearity = 0.063). A curvilinear relationship existed for participants in the non-advanced stage, with a turning point at DII = 3.451 (Table 5).

**Table 5:** Threshold effect of DII levels on Deps in participants with non-advanced CKM.

DII	β-coefficients (95%CI)	*P*-value	Log likelihood ratio test
All data	0.173 (1.127, 1.254)	<0.001	0.009
<3.451	0.127 (1.068, 1.207)	<0.001	
>=3.451	0.611 (1.335, 2.544)	<0.001	

### 3.4 Subgroup analysis and interaction testing

**Figure 5:**
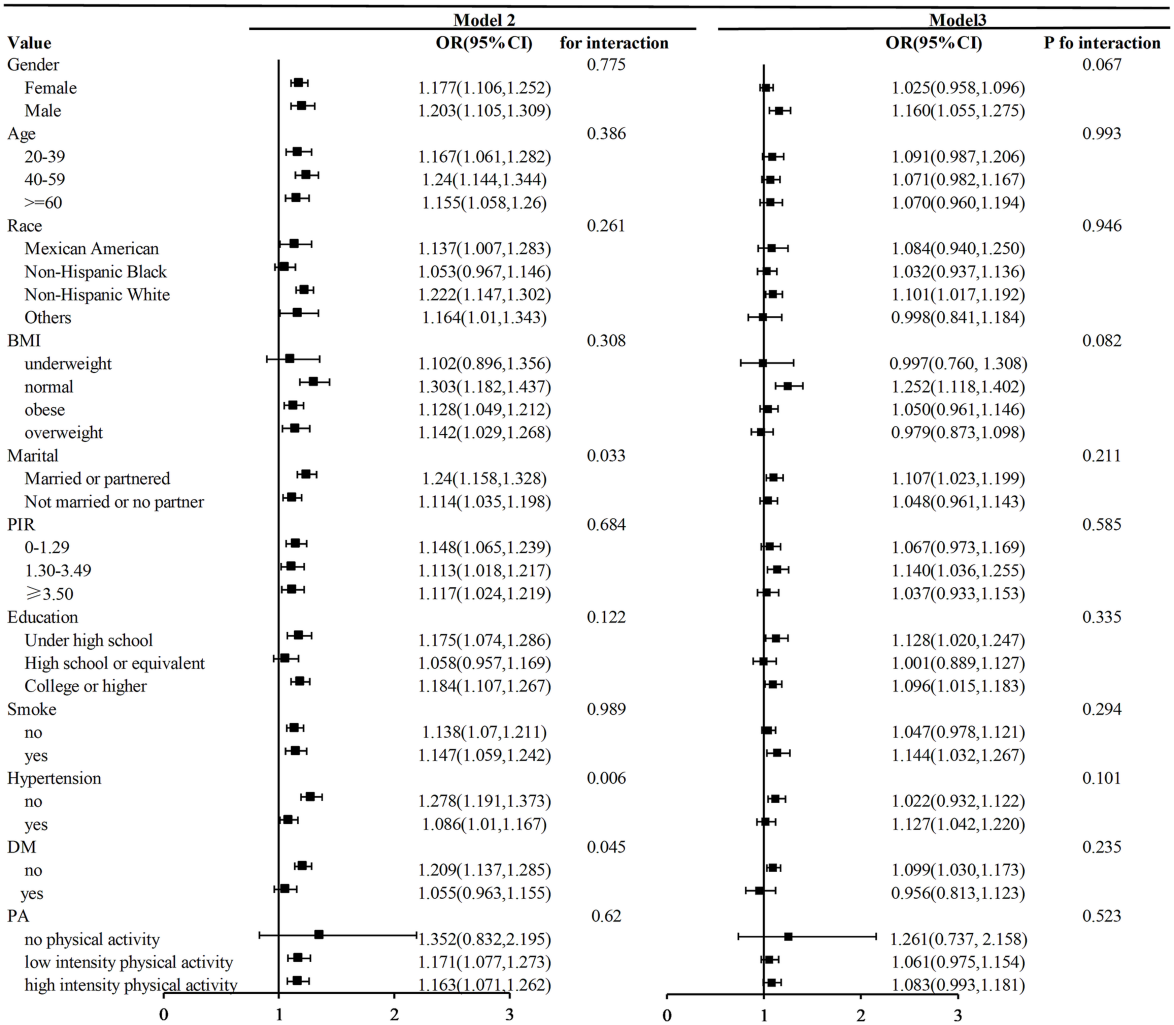
Subgroup analysis of the association between DII and Deps in participants with CKM. Model 2, adjusted for gender, age, and race. Model 3, PIR, BMI, marital status, education, diabetes, hypertension, and smoke were further adjusted based on the previous model.

In this study, we conducted subgroup analyses and interaction tests to investigate the consistency of the relationship between DII and Deps among CKM participants, stratifying participants in both the minimally and fully adjusted models (Figure 5). The results showed that this link was not significantly modified by different gender, race, age groups, BMI, marriage, smoking status, PIR, diabetes, hypertension, education level, or PA in the fully adjusted model (*P* interaction > 0.05). However, in minimally adjusted models, a significant interaction was observed for marriage, hypertension, and diabetes (*P* interaction < 0.05), suggesting that the effect of DII on Deps risk may vary by these factors. Furthermore, the positive correlation between DII and Deps remained consistent across gender, age groups, PIR, education level, smoking status, and hypertension in CKM participants, highlighting the potential applicability of this relationship in diverse population contexts.

### 3.5 Mediation effects

**Figure 6:**
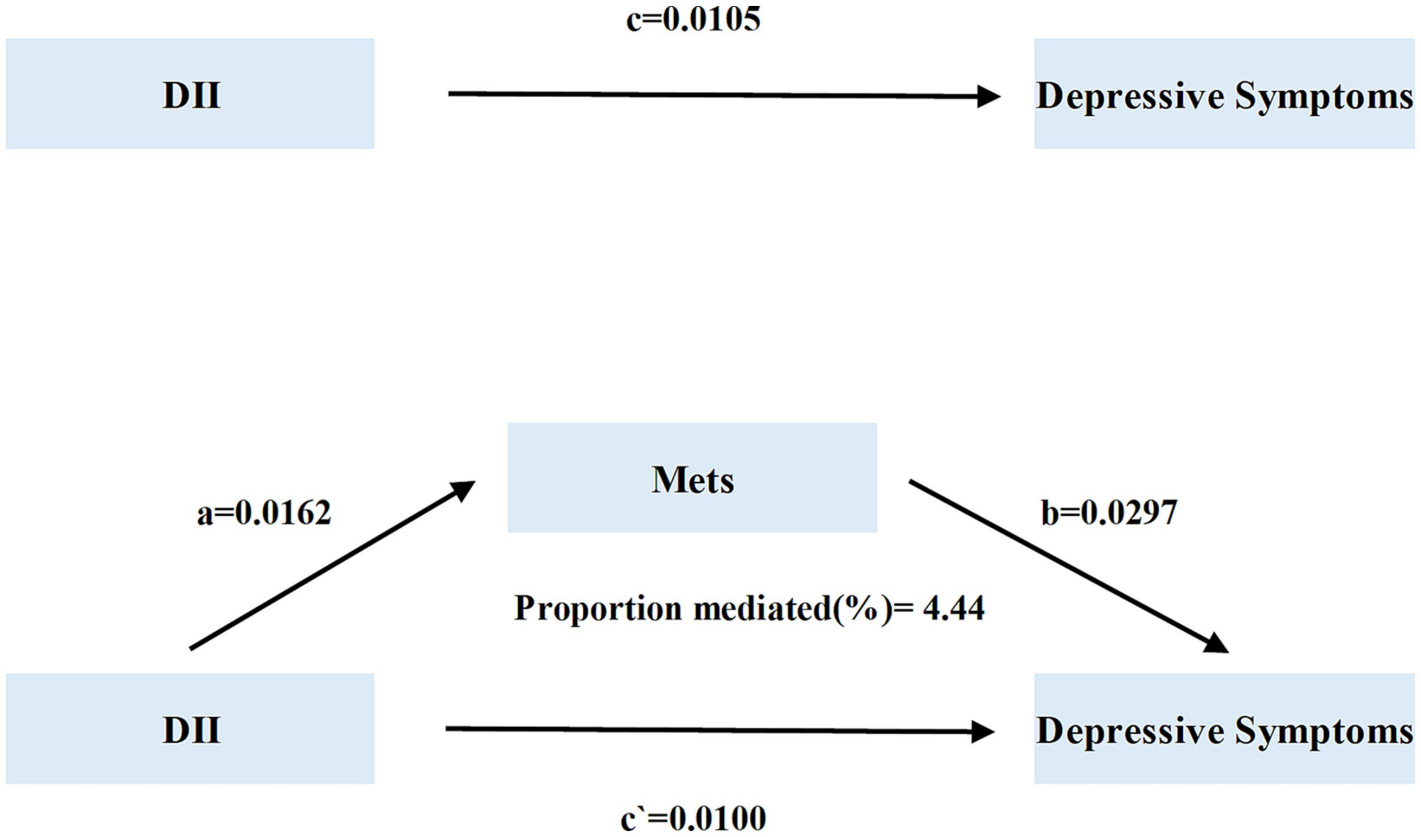
Plot of the mediation effect of Mets in DII and Deps in participants with CKM.

In the mediation model, DII level was the predictor, Mets was the mediator, and Deps was the outcome factor. The statistical analysis demonstrated that Mets exhibited a statistically significant mediating effect, accounting for 4.44% of the total effect between DII and Deps (Indirect Effect = 0.0102, *P* for mediation proportion <0.05, Table 6). The results are shown in Figure 6. The results of the Sobel–Goodman mediation test also supported the existence of a mediating effect (coefficient = 0.0005, SE = 0.001, *z* = 3.5061, *p* < 0.005).

**Table 6:** Involvement of Mets as a mediator in the associations between DII and Deps in participants with CKM.

Variables	Estimate	95% CI lower	95% CI upper	*P*-value
Total effect	0.0106	0.0080	0.0100	<0.0001
Mediation effect	0.0005	0.0003	0.0009	<0.0001
Direct effect	0.0101	0.0074	0.0100	<0.0001
Proportion mediated	0.0444		0.0800	<0.0001

### 3.6 Sensitivity analysis

Sensitivity analyses were conducted to evaluate the robustness of the relationship between DII and Deps. In the fully adjusted categorical model, there was no statistically significant association for the second tertile (T2: OR = 1.113; 95%CI, 0.760–1.630, *p* = 0.577). A positive correlation was observed in the continuous model (OR = 1.099; 95%CI, 1.023, 1.179; *p* = 0.010), while other results remained stable, even after excluding obese participants. In addition, following multiple imputations for missing covariates, the highest DII tertile was associated with a 45% higher risk of Deps compared to the lowest quartile, T3 (OR = 1.452; 95%CI, 1.178–1.789, *p* < 0.001). The results are shown in Table 7. These findings were consistent with the primary analyses, indicating that the observed relationship between DII and Deps in CKM participants is stable and reliable.

**Table 7:** Sensitivity analyses.

Characteristics		OR (95% CI)	*P*-value	*P* for trend
All participants (unweighted)	DII (Continuous)	1.065 (1.018,1.114)	0.007	–
Non-obese participants (weighted)	DII (Continuous)	1.099 (1.023,1.179)	0.010	–
	T1 (Categories)	1[Ref]	1[Ref]	0.607
	T2 (Categories)	1.113 (0.760,1.630)	0.577	
	T3 (Categories)	1.367 (1.017,1.838)	0.039	
All participants after multiple interpolation (weighted)	DII (Continuous)	1.103 (1.052,1.157)	<0.001	–
	T1 (Categories)	1[Ref]	1[Ref]	<0.001
	T2 (Categories)	1.102 (0.875,1.389)	0.406	
	T3 (Categories)	1.452 (1.178,1.789)	<0.001	

## 4 Discussion

This cross-sectional study revealed for the first time a positive correlation between DII and the prevalence of Deps in CKM patients. After adjusting for confounders, each unit increase in participants’ DII was associated with an 18.7% increase in the incidence of Deps. The study identified a J-shaped correlation between DII and Deps in CKM patients, with a segmentation effect. Notably, a curvilinear relationship existed in participants at non-advanced stages of CKM, while no significant non-linear relationship was observed in those at advanced stages. Moreover, mediation analysis indicated that Mets accounted for 4.44% of the mediating effect between DII and Deps in participants with CKM.

The CKM syndrome is a systemic disease characterized by complex pathophysiological processes, including metabolic risk factors, CKD, and CVD (3). Numerous studies have established a significant relationship between the components of CKM and Deps. A prospective cohort study from the UK Biobank has demonstrated a dose–response association between the progression of CKM syndrome and the onset of depression and anxiety. This finding suggests that treating CKM syndrome could be an effective strategy for preventing these mental health conditions, emphasizing the importance of integrated interventions and treatments.

Although our study is among the first to investigate the relationship between DII and Deps in participants with CKM, previous research has established links between DII and Deps. The OR for the association between DII and depression was 1.05 (95% CI, 1.04, 1.06), with a J-shaped relationship observed (27). Diets can be classified as either pro-inflammatory or anti-inflammatory (28). Pro-inflammatory diets lead to low-grade chronic inflammation, which increases the risk of developing depression (29). The mechanisms by which diet influences Deps are multifaceted, primarily altering neuroimmune and neuroendocrine pathways, involving the hypothalamic–pituitary–adrenal (HPA) axis and the gut-brain axis (30, 31). Consumption of a pro-inflammatory diet increases reactive oxygen species (ROS) levels, which can directly damage neurons (32). Moreover, ROS further enhances the expression of pro-inflammatory cytokines by activating inflammatory vesicles, such as NLRP3, or through the activation of transcription factors (33). When cytokines stimulate the HPA axis, the release of stress hormones, including corticotropin-releasing hormone (CRH) and cortisol, may contribute to the onset of depression (33). The gut-brain axis, a complex bidirectional communication pathway between the gut microbiota and the brain, plays a crucial role in regulating mood and cognition (30). Due to dietary influences, dysbiosis of the gut microbiota results in the excessive production of gut-derived metabolites and microbial components. When these substances pass through a compromised intestinal barrier and enter the bloodstream, they exacerbate systemic inflammation (e.g., elevated levels of IL-1β and TNF-α) (34, 35). This systemic inflammation, in turn, amplifies neuroinflammation in the brain, further worsening depressive symptom (36). Notably, siRNA-based therapies, through post-transcriptional modifications to silence specific genes, have shown potential in improving neuroimmune and neuroendocrine dysregulation, offering a promising approach for depression (37, 38).

Our findings also highlight the influence of various factors strongly associated with Deps in participants with CKM. These include female gender, non-Hispanic Black ethnicity, unmarried status or lack of a partner, higher education (college or above), obesity, low PIR, and low-intensity physical activity. Notably, the impact of the DII on Deps was more pronounced in men, which contrasts with earlier studies suggesting that women are more prone to experience Deps in the context of CKM. This may be attributed to the influence of estrogen on the prevalence of depression in women (39). Additionally, our research revealed a negative correlation between PIR and depression in individuals with CKM, potentially due to lower levels of social cohesion, healthcare access, and treatment adherence (40). This observation is consistent with existing literature, which highlights that low-income populations are more vulnerable to developing Deps (39). Although some studies have shown that higher-educated people experience better mental health (41), it is important to note that low economic returns on educational attainment may prevent well-educated individuals from fully benefiting from the mental health advantages of education (42). A survey of PhDs discovered that 45% of them had experienced depression (43).

In our study, participants in stages 1–4 had a significantly higher risk of depression compared to those in stage 0. Furthermore, individuals in advanced stages demonstrated an elevated risk of depression compared to those in non-advanced stages. CKM syndrome could be related to a greater risk of symptom burden, frailty, chronic pain, and decreased quality of life, all of which could result in increased emotional burden (44–46). Overall, given the high prevalence of depression among CKM patients, along with its negative impact on overall health and quality of life, healthcare providers should consider evaluating the DII and implementing dietary interventions to mitigate inflammation in CKM patients, which may also improve their mental health. Specific measures include a focus on nutrition counseling. Anti-inflammatory diets, such as the Mediterranean diet or plant-based dietary patterns rich in fruits, vegetables, whole grains, and omega-3 fatty acids, should be adopted. Additionally, reducing the intake of pro-inflammatory foods, including processed meats, refined carbohydrates, and excess salt, can further support mental health. Incorporating these dietary strategies into daily life and developing personalized care plans could play a crucial role in improving both the physiological and psychological outcomes of CKM patients (47). However, it is important to note that depression may increase the likelihood of CKM patients being non-adherent to dietary recommendations. Improving adherence in this subgroup is an essential factor in ensuring the successful implementation of recommended diets (48).

Through mediation analysis, our findings suggested that Mets partially mediates the relationship between diet and Deps. The potential for metabolic dysregulation to lead to neuropsychiatric outcomes in high-risk populations has been emphasized in previous studies (49, 50). A pro-inflammatory diet contributes to the onset of metabolic syndrome, with insulin resistance as its core feature. This condition affects glucose metabolism in the brain and the synthesis of neurotransmitters such as serotonin, ultimately contributing to the onset of depression. Some studies have found that individuals with insulin resistance have a higher likelihood of developing depression (51), suggesting that improving insulin resistance could be a key strategy for alleviating depressive symptoms.

The limitations of our study should not be overlooked. Firstly, due to a lack of research data, DII was calculated using 28 quantifiable food parameters. Secondly, although data collection procedures were rigorously validated through repeated measures, the operationalization of both exposure (24-h self-reported dietary recalls) and outcome variables (PHQ-9 Deps screening instrument) introduces inherent limitations through potential recall bias. Thirdly, some CKM indicators, which were self-reported, may lead to misclassification and introduce recall bias in our study. Furthermore, other CVDs, such as atrial fibrillation and peripheral artery disease, were not reported in NHANES. Fourthly, the cross-sectional design limits our ability to infer causality and establish temporality between DII and Deps with CKM. Despite our efforts to adjust for known confounders, residual or unmeasured confounding factors may persist. Lastly, since our study sample was limited to U. S. adults, the generalizability of our findings to other populations may be limited, underscoring the need for future research in diverse populations.

## 5 Conclusion

This study highlights the positive correlation between higher consumption of pro-inflammatory diets and an increased risk of Deps in participants with CKM, revealing a mediating role for the Mets. Furthermore, participants at high risk of CKM were found to be at a greater risk of developing Deps. These findings support the importance of comprehensive interventions and suggest that Deps can be prevented and reduced by promoting anti-inflammatory diets and limiting pro-inflammatory intake. Given that CKM comorbid with Deps is a multifaceted condition influenced by multiple factors, a comprehensive and personalized treatment approach is critical to address these complexities. Further cohort studies and mechanistic research are needed to explore this comorbidity.

## Data Availability

The original contributions presented in the study are included in the article/Supplementary material, further inquiries can be directed to the corresponding author.

## Footnotes

^1^ https://www.cdc.gov/nchs/nhanes/

## Glossary

DII: dietary inflammatory index
Deps: depressive symptoms
CKM: cardiovascular-kidney-metabolic
NHANES: National Health and Nutrition Examination Survey
Mets: metabolic syndrome
AHA: American Heart Association
CKD: chronic kidney disease
CVD: cardiovascular disease
WHO: The World Health Organization
CRP: C-reactive protein
BMI: body mass index
KDIGO: Kidney Disease Improving Global Outcomes
PREVENT: Predicting Risk of CVD EVENTs
PHQ-9: Patient Health Questionnaire
PIR: family income-to-poverty ratio
PA: physical activity
GAM: The generalized additive model
RCS: restricted cubic spline
DE: direct effect
IE: indirect effect
TE: total effect
HPA: hypothalamic–pituitary–adrenal
ROS: reactive oxygen species
CRH: corticotropin-releasing hormone
