# Supplementary material for: Association between the dietary inflammatory index and depressive symptoms in adults with cardiovascular–kidney–metabolic syndrome: evidence of metabolic syndrome as a mediator
Source: Front Nutr. 2025 Jul 30;12:1623482. doi: 10.3389/fnut.2025.1623482 (PMC12343276; doi:10.3389/fnut.2025.1623482)
Supplement: Supplementary file 1 [file Table_1.docx]

| Table S1. Definitions of CKM Syndrome Stages | |
| --- | --- |
| CKM Syndrome Stages | Definition |
| Stage 0 | Individuals with normal body mass index (BMI) (<23 kg/m^2^ for individuals with Asian ethnicity and <25 kg/m^2^ for other racial and ethnic groups), normal waist circumference (<80 and <90 cm for women and men with Asian race, respectively, and <88 and <102 cm for women and men in all other race and ethnicity categories, respectively) who did not meet criteria for the other stages. |
| Stage 1 | Individuals with an elevated BMI (≥23 kg/m^2^ for individuals with Asian race and >25 kg/m^2^ for all other race and ethnic groups), an elevated waist circumference (≥80 and ≥90 cm for women and men with Asian race, respectively, and ≥88 and ≥102 cm for women and men in other race and ethnicity categories, respectively), or prediabetes (defined as a glycated hemoglobin of 5.7% to <6.5% or a fasting blood glucose of 100 mg/dL to <126 mg/dL). |
| Stage 2 | Individuals with metabolic risk factors or moderate-to-high-risk CKD per Kidney Disease Improving Global Outcomes (KDIGO) criteria, as recommended by the AHA. Qualifying metabolic risk factors included elevated fasting serum triglycerides (≥135 mg/dL), hypertension, diabetes, or metabolic syndrome (≥3 of the following: elevated waist circumference, low high density lipoprotein cholesterol (HDL) level [<40 mg/dL or <50 mg/dL for men or women, respectively], fasting serum triglycerides ≥150 mg/dL, elevated blood pressure [systolic blood pressure ≥130, diastolic blood pressure ≥80 mmHg, and/or use of blood pressure-lowering medications], or prediabetes). CKD stages were identified based on GFR and urinary albumin-to-creatinine ratio. |
| Stage 3 | Individuals with very-high-risk KDIGO CKD stages or a high-predicted 10-year CVD risk. 10-year cardiovascular risk was estimated with the AHA Predicting Risk of CVD EVENTs (PREVENT) equations. High risk was defined as ≥20% 10-year CVD risk. |
| Stage 4 | Individuals based on self-reported established cardiovascular disease (coronary heart disease, angina, heart attack, heart failure, and stroke). Atrial fibrillation and peripheral artery disease were not included, as these data were not available. |

| TableS2. Category and definition of the variables | | |
| --- | --- | --- |
| Variables | Variable classification | Original data |
| Gender | Female, Male | - |
| Age | 20-39, 40-59, >=60 | - |
| Race | Mexican American | Mexican American |
|  | Non-Hispanic White | Non-Hispanic White |
|  | Non-Hispanic Black | Non-Hispanic Black |
|  | Others | Other Race - Including Multi-Racial, Other Hispanic |
| Education | Under high school | Less Than 9th Grade, 9-11th Grade (Includes 12th grade with no diploma), 9-11th grade (Includes 12th grade with no diploma), Less than 9th grade |
|  | High school or equivalent | High School Grad/GED or Equivalent, High school graduate/GED or equivalent, |
|  | College or higher | Some College or AA degree, College Graduate or above, Some college or AA degree, College graduate or above |
| PIR | 0-1.29, 1.30-3.49, ≥3.50 |  |
| BMI | Underweight | <18.5 Kg/m^2^ |
|  | Normal | 18.5-25 Kg/m^2^ |
|  | Overweight | 25-30Kg/m^2^ |
|  | Obese | >=30 Kg/m^2^ |
| Marital status | Married or partnered | Married, Married/Living with Partner, Living with partner |
|  | Not married or no partner | Never married, Divorced, Widowed, Widowed/Divorced/Separated, Separated, |
| PA | no PA | <60, MET-min/week |
|  | Low intensity PA | 60-2880, MET-min/week |
|  | High intensity PA | >2880, MET-min/week |
| Smoke | No | smoked less than 100 cigarettes in life, smoked more than 100 cigarettes in life and smoke not at all now |
|  | yes | smoked moth than 100 cigarettes in life and smoke some days or every day |
| Diabetes | No | If both of the following conditions are satisfied, it is YES, and vice versa:  1.doctor told you have diabetes  2.Use of diabetes medication or insulin, |
|  | yes |  |
| Hypertension | No | If one of the following conditions is met, it is YES, and none of them is met, it is NO:  1.doctor told you have diabetes  2. the use of antihypertensive medication  3. systolic blood pressure ≥ 140 mmHg, diastolic blood pressure ≥ 90 mmHg |
|  | Yes |  |
